# Preparation of Sorbents Containing Straetlingite Phase from Zeolitic By-Product and Their Performance for Ammonium Ion Removal

**DOI:** 10.3390/molecules26103020

**Published:** 2021-05-19

**Authors:** Agnė Mikelionienė, Danutė Vaičiukynienė, Aras Kantautas, Algirdas Radzevičius, Katarzyna Zarębska

**Affiliations:** 1 Faculty of Water and Land Management Agriculture Academy, Vytautas Magnus University, Studentu 11, LT-53361 Akademija, Lithuania; agne.mikelioniene@gmail.com (A.M.); algirdas.radzevicius@vdu.lt (A.R.); 2 Faculty of Civil Engineering and Architecture, Kaunas University of Technology, Studentu 48, LT-51367 Kaunas, Lithuania; aras.kantautas@ktu.lt; 3 Faculty of Energy and Fuels, AGH University of Science and Technology, A. Mickiewicza 30, 30-059 Kraków, Poland; katarzyna.zarebska@agh.edu.pl

**Keywords:** fluid catalytic cracking (FCC) catalysts, straetlingite, removing NH_4_^+^ ions, P sorption

## Abstract

In this study, straetlingite-based sorbents were used for NH_4_^+^ ion removal from a synthetic aqueous solution and from the wastewater of an open recirculation African catfish farming system. This study was performed using column experiments with four different filtration rates (2, 5, 10, and 15 mL/min). It was determined that breakthrough points and sorption capacity could be affected by several parameters such as flow rate and mineral composition of sorption materials. In the synthetic aqueous solution, NH_4_^+^ removal reached the highest sorption capacity, i.e., 0.341 mg/g with the S30 sorbent at a filtration rate of 10 mL/min and an initial concentration of 10 mg/L of NH_4_^+^ ions. It is important to emphasize that, in this case, the Ce/C0 ratio of 0.9 was not reached after 420 min of sorption. It was also determined that the NH_4_^+^ sorption capacity was influenced by phosphorus. In the wastewater, the NH_4_^+^ sorption capacity was almost seven times lower than that in the synthetic aqueous solution. However, it should be highlighted that the P sorption capacity reached 0.512 mg/g. According to these results, it can be concluded that straetlingite-based sorbents can be used for NH_4_^+^ ion removal from a synthetic aqueous solution, as well as for both NH_4_^+^ and P removal from industrial wastewater. In the wastewater, a significantly higher sorption capacity of the investigated sorbents was detected for P than for NH_4_^+^.

## 1. Introduction

Natural and synthetic zeolitic materials have been used for NH_4_^+^ removal from water, and there have been no data indicating that zeolite negatively affects fish life [1]. In recent years, there have been many studies related to the removal of NH_4_^+^ via an ion exchange process using clinoptilolite. Wijesinghe et al. [2] used natural zeolites for NH_4_^+^ adsorption, and determined that the NaCl treatment of natural zeolites improved the NH_4_^+^ sorption capacity by 25%; the maximal sorption capacity increased from 9.48 mg-N/g for untreated zeolites to 11.83 mg-N/g for Na^+^-treated zeolites. Aziz et al. [3] stated that thermally activated natural zeolite at 150 °C improved the NH_4_^+^ sorption capacity from 73.8% to 46.3% as compared with untreated zeolite. Sarioglu et al. [4] studied NH_4_^+^ removal by using natural zeolite based on 45% clinoptilolite, 35% mordenite, and 15% feldspar. The experiments were carried out by using untreated zeolite, as well as zeolite that was chemically treated with acid. The results showed that the highest sorption capacity with untreated zeolite was 1.08 mg NH_4_^+^-N/g when the pH was four, and the highest sorption capacity for acid-treated zeolite was 1.32 mg NH_4_^+^-N/g.

To date, many studies have been conducted using different synthetic zeolites for the removal of NH_4_^+^ from wastewater. The waste of coal fly ash biomass has been used to produce sodalite via a microwave and ultrasound irradiation method, as reported by Makgabutlane et al. [5]. New synthesized sodalite exhibited higher NH_4_^+^ removal efficiency (up 82%) from urine as compared with natural zeolite (clinoptilolite). In another study by [6], synthetic zeolite was made from electrolytic manganese residue by using fusion. In this case, NH_4_^+^ sorption capacity reached up to 27.89 mg/g when the initial NH_4_^+^ concentration was 200 mg/L at 35 °C. Zhang et al. [7] used the same fusion method to convert fly ash into a faujassite-type zeolite. This synthetic zeolite was used for NH_4_^+^ removal with 2.79 meq/g of cation exchange capacity.

Several studies have been conducted on NH_4_^+^ ion exchange using the presentation of different cations and anions. Nitrogen and P are usually the leading causes of eutrophication in the fish farming industry; therefore, the removal of NH_4_^+^ and P from wastewater has been the focus of many studies. Huang et al. [8] stated that other cations and anions had a significant influence on the removal of NH_4_^+^ using zeolites, and determined their sorption capacity order; the order of the cation solutions for removal of NH_4_^+^ was Na^+^ > K^+^ > Ca^2+^ > Mg^2+^, and the order for anion solutions was carbonate > chloride > sulfate > phosphate, at identical mass concentrations of NH_4_^+^ ions. Wu et al. [9] stated that zeolite synthesized from fly ash could be used for NH_4_^+^ and phosphate removal, and also reported that the potential of its removal capacity was not reduced at low concentrations of NH_4_^+^ and phosphate. Similar results were published by Drenkova-Tuhtan et al. [10]. They stated that the competition of P species (phosphate, phosphonates) and various metal ions such as Ca^2+^, Pb^2+^ and Cu^2+^ had significant influence on the sorption of phosphorous compounds.

NH_4_^+^ can be removed from wastewater in various ways under static (bath methods) and dynamic (column method) conditions. Sprynskyy et al. [11] investigated NH_4_^+^ ion removal by using clinoptilolite under dynamic conditions. They determined that clinoptilolite could dominate external diffusion for NH_4_^+^ sorption. Ershov et al. [12] determined that the cation exchange capacity of mordenite for NH_4_^+^ ions was 1.64 meq/g by using an initial NH_4_^+^ concentration of 1000 mg/L. In our previous study, the efficiency of NH_4_^+^ ion removal was determined for zeolitic by-product under static conditions [13].

By analyzing scientific articles related to the sorption of NH_4_^+^ ions using zeolites, we did not find information about the sorption capacity of sorbents containing straetlingite phase. These sorbents will expand the base of sorbents which is used for the sorption of ammonium ions. In this study, we aim to investigate using four different sorption materials containing straetlingite phase for NH_4_^+^ ion removal from NH_4_^+^ polluted wastewater prepared in a laboratory, and wastewater from a fish farm, and to evaluate the effects of filtration rate in synthetic solutions and wastewater on NH_4_^+^ and P removal.

## 2. Results and Discussions

### 2.1. Mineral Composition of Sorption Materials

**Figure 1:**
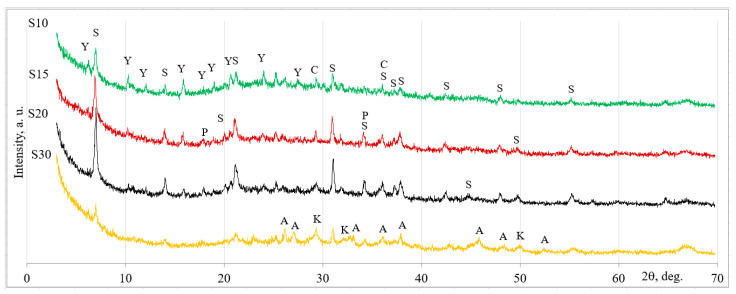
X-ray diffraction patterns of hardened zeolitic materials after 7 days. S, straetlingite Ca_2_Al((AlSi)_1.11_O_2_)(OH)_12_(H_2_O)_2.25_ (80–1579); P, portlandite Ca(OH)_2_ (44–1481); A, aragonite CaCO_3_ (3–893); K, calcium silicate hydrate Ca_1.5_Si O_3.5_∙*x* H_2_O (33–306); C, cal-cite CaCO_3_ (72–837); Y, faujasite Al_60.352_∙Si_139_∙O_371.52_∙H_5.984_ (73–2313).

The mineral composition of sorption materials was evaluated using X-ray diffraction (XRD) analysis (Figure 1). The amount of CaO used had a significant effect on the mineral composition of the sorption materials. The cementitious compounds, i.e., straetlingite and calcite, formed in the S10 sorbent, with a similar amount of faujasite, were left unreacted. When a larger amount of CaO (S15 sorbent) was incorporated into the system, the peak intensities assigned to straetlingite were slightly increased. Additionally, calcite, faujasite, and portlandite were detected. By increasing CaO to 20% (S20 sorbent), the cementitious compound straetlingite had significantly higher peak intensities, and small amounts of calcite, faujasite, and portlandite were found. So, it is possible to state that the highest amount of straetlingite formed when the mixture was composed of 20% CaO. CaO of 30% completely changed the mineral composition of the sorption material of the S30 sorbent. In this case, cementitious compounds calcium silicate hydrate, aragonite, and calcite were formed after 7 days of reaction. The zeolitic by-product was a pozzolanic material, since faujasite and portlandite were also detected.

Our findings are in agreement with the findings of other studies on the formation of straetlingite, including the following: Heikal et al. [14] reported that straetlingite (Ca_2_Al((AlSi)_1.11_O_2_)(OH)_12_(H_2_O)_2.25_) formed in blends of calcium aluminate cement and ground granulated blast furnace slag in the presence of moisture, and that the optimal temperature for this reaction was 20–30 °C; Yaman et al. [15] determined that the formation of straetlingite positively influenced the compressive strength of sorbents; Xu et al. [16] proposed that the formation of straetlingite in Portland-based concrete with the addition of fly ash could explain the improved mechanical properties; Straetlingite has been formed in a pozzolanic admixture based on metakaolin and Ca(OH)_2_ at curing temperature of 20 °C after 3 days [17]; Frıas et al. [18] stated that the main phases which form during the pozzolanic reaction between metakaolin and lime at an ambient temperature are calcium silicate hydrate, calcium aluminum hydrate, and calcium aluminum silicate hydrate (straetlingite).

### 2.2. Microstructure of Sorption Materials

**Figure 2:**
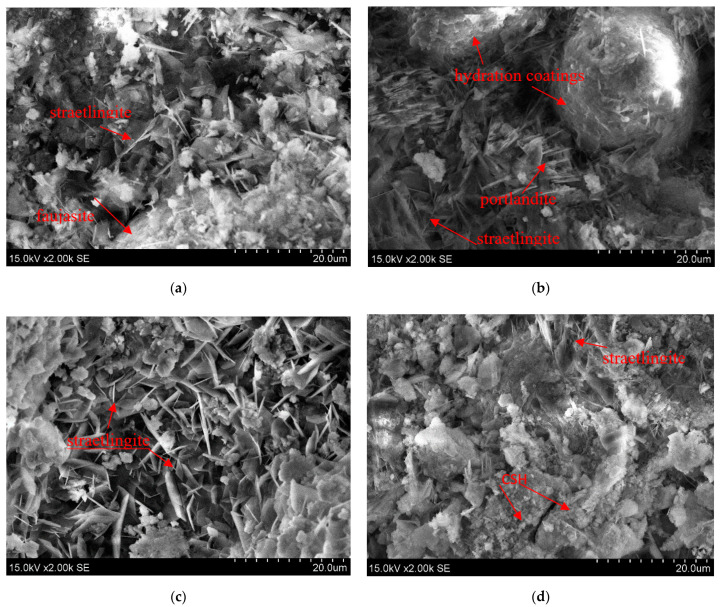
SEM images of the sorption materials made with zeolitic by-product and CaO: (**a**) S10; (**b**) S15; (**c**) S20; (**d**) S30.

The microstructures of the sorption materials formed during the chemical reactions between the solid compounds zeolitic by-product and CaO, with the addition of water, are shown in Figure 2. The mineral compositions of the sorption materials were confirmed by SEM analysis. The morphology of the hydration products had a strong influence on the amounts of solid materials such as zeolitic by-product and CaO.

In S10 sorbent, typical hexagonal crystals of straetlingite were dominant [19]. Moreover, spherical particles of faujasite coated with hydration products such as amorphous calcium silicate hydrate were also detected [20].

During hydration, the microstructure of the sorption material made from higher amounts of CaO (15%) and zeolitic by-product (S20 sorbent) consisted of spherical particles of faujasite coated with hydration products, column aggregate shaped portlandite [21], and hexagonal particles of straetlingite.

Ma et al. [22] stated that straetlingite (C_2_ASH_8_) is also known as hydrated gehlenite. In S20 sorbent, hexagonal platelets were dominant in the hydration products of zeolitic by-product and lime. This morphology of the hydrates could be assigned to straetlingite [23].

During hydration, the S30 sorbent, which was made from 70% zeolitic by-product and 30% CaO, produced calcium silicate hydrate (CSH) semi-amorphous conglomerate [24]. In these CSH semi-amorphous conglomerates, hexagonal platelets of straetlingite crystals were incorporated during the hydration reactions.

Therefore, in all investigated sorption materials, straetlingite formed typical hexagonal crystals, and the morphology of CSH was semi-amorphous conglomerates in the system of hydration products.

### 2.3. The Sorption of NH_4_^+^ Ions by Straetlingite-Based Sorbents in a Synthetic Aqueous Solution

**Figure 3:**
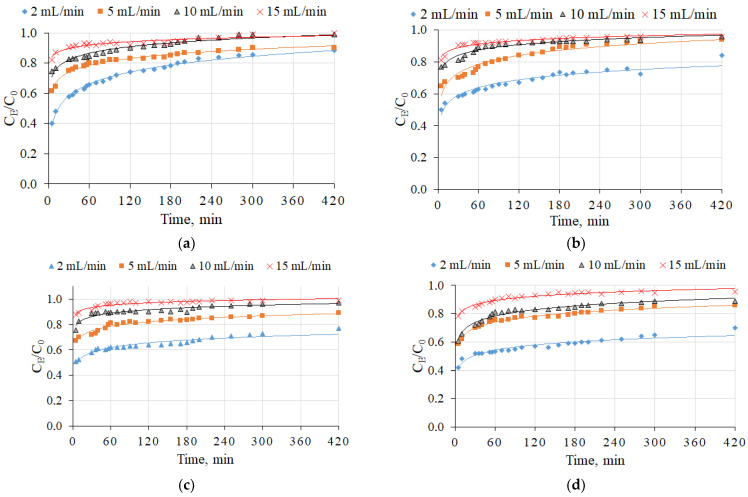
The influence of the flow rate on the breakthrough curves for NH_4_^+^ ion sorption by straetlingite-based sorbents in a synthetic aqueous solution: (**a**) S10 (according to the Table 2); (**b**) S15; (**c**) S20; (**d**) S30. The initial NH_4_^+^ ion concentration in the synthetic aqueous solution was 10 mg/L and the filtration rates were 2, 5, 10, and 15 mL/min.

**Figure 4:**
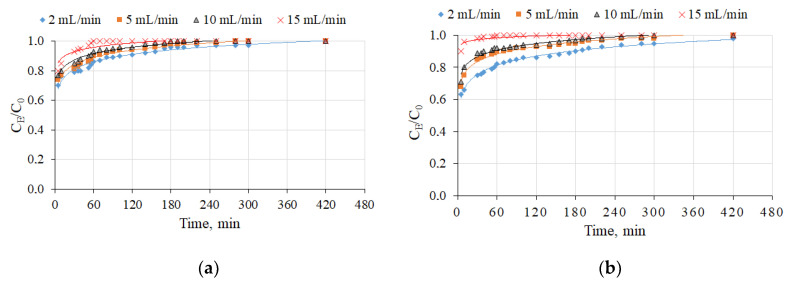
Influence of the flow rate on the breakthrough curves of NH_4_^+^ ion sorption by straetlingite-based sorbents (S20 and S30) in wastewater from an open recirculation African catfish farming system: (**a**) S20(according to the Table 2); (**b**) S30. The initial NH_4_^+^ ion concentration in the synthetic aqueous solution was 10 mg/L and the filtration rates were 2, 5, 10, and 15 mL/min.

In the first part of this study, a synthetic aqueous solution was used for the evaluation of NH_4_^+^ ion removal using four types of straetlingite-based sorbents (sorbents S10, S15, S20, and S30). Figure 3 shows the results of NH_4_^+^ breakthrough curves at four filtration rates (2, 5, 10, and 15 mL/min). The initial NH_4_^+^ ion concentration was the same in all experiments (10 mg/L). The experiments were continued until the sorbents reached saturation value (i.e., C_E_/C_0_ = 1). The obtained breakthrough curves are shown in Figure 4. The breakthrough point times were at 420, 280, 120, and 20 min for 2, 5, 10, and 15 mL/min, respectively (S10 sorption material). For the S15, S20, and S30 sorbents, the breaking point times were longer than 420 min, because after 420 min the C_E_/C_0_ ratio was less than 0.9 (Figure 3). Therefore, when the filtration rate increased, the breakthrough times for all investigated sorption materials were reduced, as shown in Table 1. Temel et al. [25] reported a similar finding and detected a breakthrough point time (90 min) with a filtration rate of 10 mL/min. The reduction in breakthrough point time could have been related to the contact time of water and sorption material; by increasing the filtration time, the contact time was decreased [26].

The filtration rate through the column had a significant influence on the NH_4_^+^ sorption capacity. Higher NH_4_^+^ sorption was obtained for the experiment with a lower flow rate, i.e., 2 mL/min. When the filtration rate through the sorption materials increased, the NH_4_^+^ sorption capacity gradually decreased (Table 1). This relationship was determined for all sorption materials that were used in this experiment. A similar relationship has been reported by Li et al. [26].

The sorption capacity and the values of breakthrough points are closely related to the filtration rate and also to the mineral composition of sorption materials. The highest sorption capacities were determined for the S30 sorbent at all of the filtration rates, and the two dominant minerals in the sorption material which acted as sorbent were straetlingite and calcium silicate hydrate. The synergetic effect of these two compounds could be the reason for the highest sorption capacity reached, which was 0.341 mg/g. In addition, Li et al. [27] reported that CSH and zeolite had significant impacts on NH_4_^+^ and P removal.

### 2.4. The Sorption of NH_4_^+^ Ions by Straetlingite-Based Sorbents in Wastewater from an Open Recirculation African Catfish Farming System

In the second part of this study, wastewater from an open recirculation African catfish farming system was used. Real wastewaters are complex solutions, as stated by Karapınar et al. [28]; instead of NH_4_^+^ ions, P was detected in the wastewater. Two of the best sorption materials, i.e., S20 and S30, were selected for these experiments on NH_4_^+^ and P removal, according to the sorption experiments using a synthetic aqueous solution (Section 2.3).

The relationship of flow rates on the breakthrough curve of NH_4_^+^ ion sorption is shown in Figure 4 and Table 2. P had a negative impact on NH_4_^+^ removal using straetlingite-based sorbents; in this case, the breakthrough point times became significantly shorter as compared with the breakthrough point times in the synthetic aqueous solution without phosphorus. The NH_4_^+^ sorption capacity decreased almost seven times when this wastewater instead of synthetic NH_4_^+^ solutions was filtered through the column.

According to Hedström et al. [29], the NH_4_^+^ sorption capacity of wastewater at breakthrough point time was about 50% lower as compared with the sorption capacity of synthetic solutions, and a similar tendency was determined by Huang et al. [8]. This decrease in NH_4_^+^ ion sorption capacity in the wastewater could be related to the completion effect of different compounds (phosphorus) found in the wastewater. Li et al. [6] determined that NH_4_^+^ sorption capacity was decreased by increasing PO_4_^3−^ anions in a system; the NH_4_^+^ sorption capacity decreased from 16.62 to 10.98 mg/g by increasing the phosphate ions from 50 mg/L to 250 mg/L. Karapınar et al. [28] used natural zeolite (Type C, Zeolith, Germany) for the removal of NH_4_^+^ and for the precipitation of calcium phosphate. Their results showed that the sorption capacity of NH_4_^+^ using zeolites decreased in the presence of phosphorus. Mazloomi et al. [30] reported that NH_4_^+^ sorption capacity decreased from about 96% to 66.5% when P anions were added into a system. This decreased NH_4_^+^ sorption capacity could be related to the presence of competing ions in the system [7]. The P anions may increase the surface tension of the aqueous phase, thereby reducing NH_4_^+^ access to the micropores and macropores of the zeolite [30].

**Figure 5:**
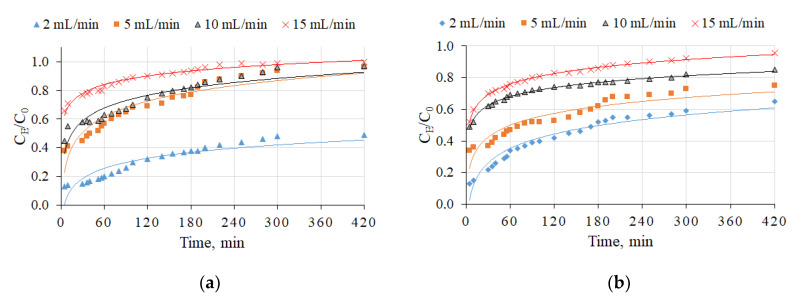
Influence of the flow rate on the breakthrough curves of total P ion sorption by straetlingite-based sorbents in wastewater from an open recirculation African catfish farming system: (**a**) S20 (according to the Table 2); (**b**) S30. The initial NH_4_^+^ ion concentration in the synthetic aqueous solution was 10 mg/L and the filtration rates were 2, 5, 10, and 15 mL/min.

In this study, straetlingite-based sorbents for the removal of total P were also evaluated. Figure 5 shows the breakthrough curves of P ion sorption.

Similar characteristics for the breakthrough curves of NH_4_^+^ ions were determined for P ions, i.e., by increasing the sorption rate, the breakthrough point times became shorter for both S20 and S30 sorption materials (Table 3). However, a C_E_/C_0_ ratio of 0.9 was not attained after 420 min of the column experiments, see Figure 5.

A longer breakthrough point time occurred with the S30 sorption material than the S20 sorption material, due to the differences in mineral compositions of the sorption materials. Straetlingite prevailed in the S20 sorbent. Meanwhile, two types of minerals, i.e., straetlingite and calcium silicate hydrate (which could act as sorption compounds) dominated in the S30 sorbent. The maximal amount of NH_4_^+^ adsorbed by the straetlingite-based sorbents was 0.0452 mg/g in the presence of phosphorus, in which the sorption capacity reached 0.512 mg/g when the initial concentration of NH_4_^+^ was 11.2 mg/L, and the sorption capacity of P was 47.0 mg/L in the wastewater from an open recirculation African catfish farming system.

The P removal was improved by using a filtration rate of 10 mL/min and the S30 sorption material based on straetlingite and calcium silicate hydrate. In this case, the sorption capacity of P reached 0.512 mg/g. Our sorption capacity values were mostly in agreement with the values reported in related studies by Chen et al. [31] and Jiang et al. [32]. Taken together, on the basis of these findings, it can be concluded that straetlingite-based sorbents are suitable for the removal of NH_4_^+^ from aqueous solutions, and especially from wastewater in which P is present.

## 3. Materials and Methods

### 3.1. Materials

For this study, reagent CaO (Chempur, Piekary Śląskie, Poland) was used with a purity of 99.0%.

Spent fluid catalytic cracking catalyst (zeolitic by-product) was received from a petroleum plant. Zeolites are commonly used in the process of fluid catalytic cracking. During a catalytic cracking process zeolite loses its catalytical properties, becomes degraded and becomes waste (by-product). The composition of these catalysts depends on the manufacturer and on the process that is going to be used. The chemical composition of the zeolitic by-product is shown in Table 4. This material can be classified as aluminosilicate material because it is based on 84% SiO_2_ + Al_2_O_3_.

**Figure 6:**
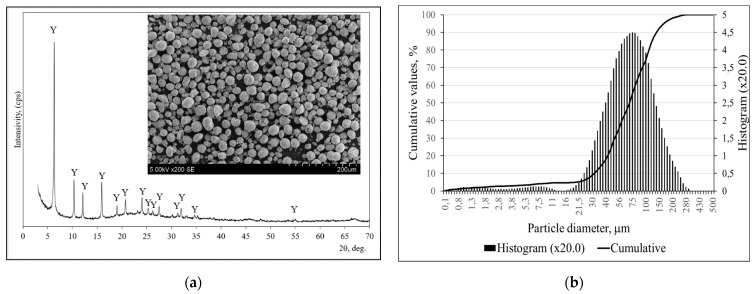
X-ray diffraction pattern with microstructure of the zeolitic by-product (**a**) and particle size distributions; (**b**). Note, Y is faujasite Al_60.352_∙Si_139_∙O_371.52_∙H_5.984_ (73-2313).

X-ray diffraction analysis was applied to determine the mineral composition of the zeolitic by-product (Figure 6a); faujasite-type zeolite was the dominant component in the by-product [33].

The microstructure of zeolitic by-product was evaluated by SEM analysis (Figure 6a) and revealed that round shaped particles were dominant in the zeolitic by-product (Figure 6b); dos Santos et al. [34] determined a similar shape of zeolitic by-product particles.

Figure 6b shows the particle size distribution curves of the zeolitic by-product used in this work, forming a peak from 16 to 280 µm, with a mean diameter of 78.39 µm.

### 3.2. The Preparation of Sorption Materials

**Figure 7:**
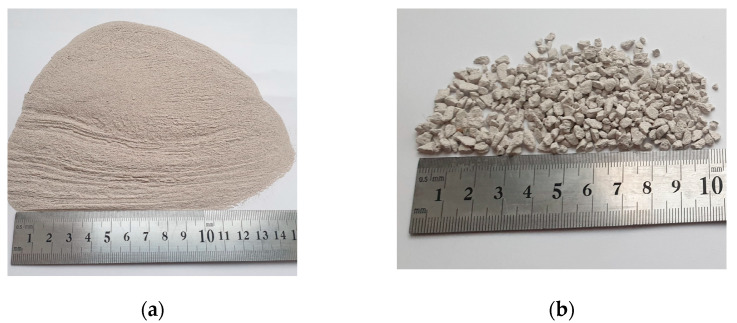
Photograph of the: (**a**) zeolitic by-product; (**b**) coarser fraction of sorption material based on straetlingite.

The sorption material granules were prepared from zeolitic by-product powder (Figure 7a) and CaO.

First, mixtures of zeolitic by-product and CaO were prepared. The compositions of the initial sorption materials based on straetlingite are shown in Table 5. There were four mixtures of sorption materials consisting of different ratios of zeolitic by-product to reagent CaO. Then, water was added, and the mixtures were mixed thoroughly. The ratio of water and solid materials in all of the sorbent samples was almost the same. Finally, the mixtures were left for 7 days of hydration; the mixtures were covered with plastic material to protect from water evaporation.

During hydration, reactions between solid compounds of zeolitic by-product and CaO, as well as water, occurred. These chemical reactions led to setting and hardening of the zeolitic by-product/ CaO /water mixtures. After 7 days, the mixtures were crushed and sifted through a sieve. For the sorption experiment, 2–4 mm sized particles were used (Figure 7b).

Two types of initial solutions were used. The first NH_4_^+^ ion initial solution was prepared by using the salt of NH_4_^+^ chloride (synthetic aqueous solution) and deionized water. The initial NH_4_^+^ ion concentration was 10 mg/L. The second initial solution (wastewater) was taken from an open recirculation African catfish farming system; in this wastewater, the NH_4_^+^ ion concentration was 11.2 mg/L and the total P concentration reached 47.0 mg/L (Table 6).

The amount of NH_4_^+^ or P in the solid phase (Q, mg/g) was calculated according to Equation (1) as follows:

(1) $$Q=\vartheta A\int_{0}^{t}\left(C_{0}-C_{E}\right)\mathrm{tdt},$$

where C_0_ and C_E_ are the influent and effluent NH_4_^+^ ion concentrations, respectively (at breakthrough point t) (mg/L); ν represents the solution filtration rate through a sorption material (m/h); A is the cross-sectional area of the column (m^2^); and t is time (h).

The breakthrough point was assessed to be when the ratio between the initial concentration and the final concentration in the inflow was 0.9 (C_E_/C_0_ = 0.9) [26]. The sorption of NH_4_^+^ at the breakthrough point was defined as its sorption capacity.

During NH_4_^+^ ion sorption from the synthetic aqueous solution, straetlingite attracts NH_4_^+^ ions from the solutions and releases calcium cations to the solutions. The ion exchange mechanism between straetlingite and the NH_4_^+^ ions from water solutions can be represented by Equation (2):

straetlingite − Ca^2+^ + nNH_4_^+^ ⇆ straetlingite − nNH_4_^+^ + Ca^2+^ (2)

This equation is similar for all zeolites (straetlingite) that have been used for the NH_4_^+^ removal [35].

### 3.3. Experimental Techniques

The chemical composition of the zeolitic by-product was determined by X-ray fluorescence using a Bruker X-ray S8 Tiger WD spectrometer (Karlsruhe, Germany), with a rhodium (Rh) tube, an anode voltage Ua up to 60 kV, and electric current I up to 130 mA. The pressed sorbent samples were measured in a helium atmosphere. Measurements were performed following the SPECTRA Plus QUANT EXPRESS method [36].

The X-ray diffraction analysis (XRD) of the materials was performed using a D8 Advance diffractometer (Bruker AXS, Karlsruhe, Germany) operating at a tube voltage of 40 kV and tube current of 40 mA. The X-ray beam was filtered with Ni 0.02 mm filter to select the Cu Kα wavelength. The sorbent samples were scanned over the range 2θ = 3–70° at a scanning speed of 6° min^−1^ using a coupled two theta/theta scan type [37].

The particle size distribution of the zeolitic by-product was determined by using a particle size laser analyzer (CILAS 1090 LD, Orleans, France). The distribution of solid particles in the air stream was 12–15 wt.%; compressed air (2500 mbar) was used as a dispersing phase; and the measuring time was 15 s [38].

The microstructures of tree-type zeolitic by-product and hardened cement pastes were studied by scanning electron microscopy (SEM) using a high-resolution scanning electron microscope (ZEISS EVO MA10, Edmonton, Canada) [39].

The solution pH was determined using a Hanna ISE pH meter (Nușfalău, Romania).

**Figure 8:**
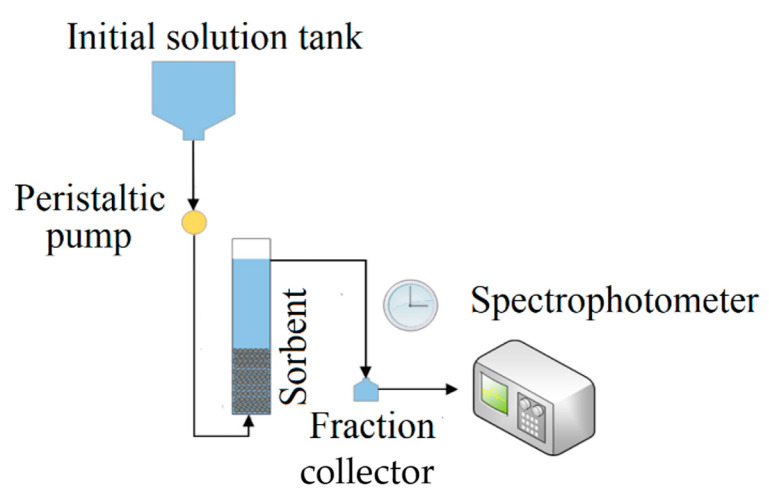
The scheme of the experimental setup for the sorption experiments.

Column tests were carried out using glass columns with a 3 cm inner diameter and 40 cm height. The zeolite bed was 6.5 cm high with a volume of 45.9 cm^3^. The experimental scheme of sorption in dynamic conditions is shown in Figure 8. The glass column was filled with different types of straetlingite-based zeolite, each with a mass of 20 g for the column tests.

A peristaltic volumetric DF-12M infusion pump (Viltechmeda, Vilnius Lithuania) was applied for the column solutions with sorption materials, at four different flow rates, i.e., 2, 5, 10 and 15 mL/min. During the sorption process, the exit solutions (effluent) were analyzed periodically. The NH_4_^+^ concentration was determined following the Nessler method [40]. The total amount of P in the solutions was evaluated according to the vanadomolybdophosphoric acid method. An HI83399 multiparameter photometer (Hanna Instruments, Nușfalău, Romania) was used for the evaluation of NH_4_^+^ and total P in the solutions. The experiment for the amount of NH_4_^+^ or phosphorous was repeated at least three times. The mean value of the triple analysis was used to calculate the amount of NH_4_^+^ or phosphorous in solution, and the limit of error for the samples was lower than 5%.

## 4. Conclusions

According to the XRD analysis, hydration reactions between zeolitic by-product and CaO resulted in the formation of straetlingite (Ca_2_Al((AlSi)_1.11_O_2_)(OH)_12_(H_2_O)_2.25_) after seven days. We determined that the NH_4_^+^ sorption capacities depended on certain factors such as the filtration flow rate and the mineral composition of sorbents. The NH_4_^+^ removal efficiency breakthrough times of all investigated straetlingite-based sorbents were reduced when the filtration rate was increased from 2 mL/min to 10 mL/min. The highest sorption capacity in a synthetic aqueous solution (i.e., 0.341 mg/g) was obtained by using the S30 sorbent with a filtration rate of 10 mL/min and an initial NH_4_^+^ ion concentration of 10 mg/L; it is important to emphasize that, in this case, a C_E_/C_0_ ratio of 0.9 was not reached after 420 min of sorption. The results for NH_4_^+^ sorption in wastewater showed an almost seven times lower sorption capacity than that in the synthetic aqueous solution; however, it should be emphasized that a P sorption capacity of 0.512 mg/g was reached. The NH_4_^+^ removal experimental results indicate that NH_4_^+^ ions can be removed from a synthetic aqueous solution, and also from the wastewater. In the case of wastewater, the P sorption capacity of straetlingite-based sorbents was significantly higher than their NH_4_^+^ sorption capacity.

## Figures and Tables

**Table 1:** The influence of filtration rate on the breakthrough point time (min) and NH_4_^+^ sorption capacity (mg/g).

Sorption Material	Filtration Rate, mL/min							
	2	5	10	15	2	5	10	15
	Breakthrough Point, min				Sorption Capacity, mg/g			
S10	420	280	120	20	0.135	0.129	0.103	0.0446
S15	>420 *	200	60	28	0.123 **	0.104	0.0694	0.0484
S20	>420 *	>420 *	52	22	0.137 **	0.178 **	0.0469	0.0404
S30	>420 *	>420 *	>420 *	60	0.169 **	0.215 **	0.341 **	0.0841

* After 420 min of sorption, a C_E_/C_0_ ratio of 0.9 was not reached. ** The sorption capacity was evaluated after 420 min of sorption.

**Table 2:** The influence of filtration rates on breakthrough point times (min) and NH_4_^+^ sorption capacities (mg/g).

Sorption Material	Filtration Rate, mL/min							
	2	5	10	15	2	5	10	15
	Breakthrough Point, min				Sorption Capacity, mg/g			
S20	100	60	52	20	0.0241	0.0313	0.0489	0.0273
S30	180	60	40	5	0.0255	0.0325	0.0452	0.0225

**Table 3:** The influence of filtration rates on breakthrough point times (min) and P sorption capacities (mg/g).

Sorption Material	Filtration Rate, mL/min							
	2	5	10	15	2	5	10	15
	Breakthrough Point, min				Sorption Capacity, mg/g			
S20	>420 *	250	262	120	0.264 **	0.2	0.341	0.181
S30	>420 *	>420 *	>420 *	250	0.215 **	0.403 **	0.512 **	0.383

* After 420 min of sorption, a *C_E_*/*C*_0_ ratio of 0.9 was not reached. ** The sorption capacity was evaluated after 420 min of sorption.

**Table 4:** Oxide composition of the zeolitic by-product (%), according to X-ray fluorescence.

SiO_2_	Al_2_O_3_	Fe_2_O_3_	La_2_O_3_	TiO_2_	MgO	CaO	Na_2_O	Cl	P_2_O_5_	SO_3_	Other
35.4	48.77	1.02	1.63	3.57	0.44	0.37	0.31	2.57	0.08	0.07	5.77

**Table 5:** The composition of the initial material mixtures.

Mixture	Zeolitic By-Product (wt. %)	Reagent CaO (wt. %)	Water and Solid Materials Ratio (W/S)
S10	90	10	0.73
S15	85	15	0.70
S20	80	20	0.70
S30	70	30	0.70

**Table 6:** Physical and chemical parameters of the wastewater from an open recirculation African catfish farming system.

Parameters and Unites	Values	Parameters and Unites	Values
Oxygen saturation	>40	NH_4_^+^, mg/L	11.2
pH	7.6	Nitrite, mg/L	<1.1
Temperature, °C	22	Nitrate, mg/L	<61
Free CO_2_, mg CO_2_/L	25	Iron, Fe, mg/L	<1.1
Total nitrogen, mg/L	<1.1	Total phosphorus, mg/L	47.0
